# Changing patterns in the association between regional socio-economic context and dental caries experience according to gender and age: A multilevel study in Korean adults

**DOI:** 10.1186/1476-072X-11-30

**Published:** 2012-07-28

**Authors:** Hoo-Yeon Lee, Youn-Hee Choi, Hyoung Wook Park, Sang Gyu Lee

**Affiliations:** 1 Department of Social Medicine, College of Medicine, Dankook University, Cheonan, South Korea; 2 Department of Preventive Dentistry, School of Dentistry, Kyungpook National University, Daegu, South Korea; 3 Department of Preventive Medicine, College of Medicine, Dankook University, 201, Manghyang-ro, Dongnam-gu, Cheonan-si, Choongnam, 330-714, South Korea

**Keywords:** Dental caries, Contextual factor, Multilevel analysis, Gender, Age

## Abstract

**Background:**

Little is known about the effects of socio-environmental factors on dental caries in different demographic situations in Asian populations. We investigated whether the nature of the association between regional socio-economic context and dental caries experience differed according to gender and age groups in Korean adults.

**Methods:**

We obtained a linked data set containing individual information from the 2000 Korean National Oral Health Survey and regional information from the “Major statistical indices of Si-Gun-Gu” (city-county-ward), published by the Korean Statistical Office. We stratified participants into women and men and into four 10-year-interval age groups (19–34, 35–44, 45–54, and 55–64 years) and analysed the linked data using a multilevel analysis. In total, 5,259 individuals were included in the final study population.

**Results:**

Regional socio-economic context was significantly associated with dental caries experience in men, but not in women. The patterns of the association between regional contextual variables and dental caries experience differed among age groups. People 35–44 years of age living in areas less dependent on the manufacturing industry and those 45–54 years of age living in areas where local government was relatively poor were more prone to have caries experience.

**Conclusions:**

The results of this study indicated that socio-economic factors affecting residents’ dental health status may operate through different mechanisms or degrees according to geographic location, suggesting that some gender- and age-defined subgroups may be likely to benefit from different types of intervention, including the development of specific health policies.

## Background

Dental caries is one of the most common health problems in developing and developed countries; epidemiological data from many countries have clearly demonstrated the high prevalence of this disease [1]. Many models have been developed to explain the aetiology and biological causes of caries; however, current models are inadequate to explain variations in caries experience among populations [2-4]. Several recent studies have examined the relationships between regional contextual factors and dental caries, finding that socio-economic context has a significant effect on the prevalence of the disease [2,3,5-9]. However, very limited information is available on these associations among subgroups of the population, especially among more vulnerable groups. For example, gerontological research findings have suggested that the regional environment may be more important to the lives of older adults than to those of younger adults, and that the health of poorer populations is more susceptible to socio-economic environmental factors [10].

The association between regional socio-economic context and dental caries experience may be characterised by various patterns based on individual characteristics, such as gender or age [2,3,7]. Biological (genetics, hormones, reproductive history) and anthropological (behavioural) factors, such as culture-based division of labour and gender-based dietary preferences, may also play roles [11,12]. Additionally, despite the large number of studies that have examined neighbourhood influences on individual oral health outcomes, few reported studies have been conducted in Asian populations [13]. Neighbourhood effects may differ between Asian and Western societies due to differences in social relationships, community formation, and economic development, as well as in individual demographic factors. In this study, we investigated whether the nature of the association between regional socio-economic context and dental caries experience differed according to gender and age groups using a representative sample of Korean adults.

## Results

Men accounted for 41.7 % and individuals aged 19–34 years accounted for 31.8 % of the 5,259 participants in the study population. Of the participants, 88.1 % had decayed, missing, and filled teeth (DMFT) index scores ≥ 1. Dental caries experience differed significantly among groups with regard to gender, age, monthly family income, regular dental clinic visitation, and factor I (degree of urbanisation; Table 1). The prevalence of caries experience was significantly higher in women than in men. The rates of caries experience increased progressively from the young adult age group (19–34 years) to the older age group (55–64 years) in both women and men. Individuals with higher family incomes were more likely to have lower caries experience rates. The prevalence of caries experience also increased progressively from the highest to lowest degree of urbanisation.

**Table 1:** Distribution of individual and regional characteristics by dental caries experience

		**Total**		**No (DMFT = 0)**		**Yes (DMFT ≥ 1)**		*p*-value*
		**No.**	**(%)**	**No.**	**(%)**	**No.**	**(%)**	
Total**		5,259	(100.0)	625	(11.9)	4,634	(88.1)	
Individual level								
Gender								
	Men	2,191	(41.7)	367	(16.8)	1,824	(83.3)	<0.0001
	Women	3,068	(58.3)	258	(8.4)	2,810	(91.6)	
Age (years)								
	19–34	1,673	(31.8)	258	(15.4)	1,415	(84.6)	<0.0001
	35–44	1,296	(24.6)	184	(14.2)	1,112	(85.8)	
	45–54	1,084	(20.6)	105	(9.7)	979	(90.3)	
	55–64	1,206	(22.9)	78	(6.5)	1,128	(93.5)	
Monthly family income (thousand Korean won)								
	<500	706	(14.0)	61	(8.6)	645	(91.4)	0.012
	500–990	1,196	(23.7)	144	(12.0)	1,052	(88.0)	
	1000–1490	1,093	(21.6)	130	(11.9)	963	(88.1)	
	≥1500	2,061	(40.8)	275	(13.3)	1,786	(86.7)	
Tooth-brushing frequency (number/day)								
	0–1	482	(9.2)	47	(9.8)	435	(90.3)	0.313
	2	2,908	(55.3)	349	(12.0)	2,559	(88.0)	
	≥3	1,867	(35.5)	228	(12.2)	1,639	(87.8)	
Regular dental clinic visits								
	No	4,756	(90.4)	541	(11.4)	4,215	(88.6)	0.000
	Yes	503	(9.6)	84	(16.7)	419	(83.3)	
Regional level								
Factor I (degree of urbanisation)								
	highest	1,255	(23.9)	158	(12.6)	1,097	(87.4)	0.024
	high	1,562	(29.7)	190	(12.2)	1,372	(87.8)	
	middle	1,184	(22.5)	155	(13.1)	1,029	(86.9)	
	low	731	(13.9)	81	(11.1)	650	(88.9)	
	lowest	527	(10.0)	41	(7.8)	486	(92.2)	
Factor II (density of services and medical facilities)								
	highest	1,114	(21.2)	141	(12.7)	973	(87.3)	0.087
	high	989	(18.8)	128	(12.9)	861	(87.1)	
	middle	1,015	(19.3)	96	(9.5)	919	(90.5)	
	low	843	(16.0)	108	(12.8)	735	(87.2)	
	lowest	1,298	(24.7)	152	(11.7)	1,146	(88.3)	
Factor III (dependence on manufacturing industry)								
	highest	1,223	(26.9)	192	(13.6)	1,223	(86.4)	0.102
	high	821	(17.8)	117	(12.5)	821	(87.5)	
	middle	857	(18.4)	112	(11.6)	857	(88.4)	
	low	600	(12.8)	73	(10.9)	600	(89.2)	
	lowest	1,133	(24.0)	131	(10.4)	1,133	(89.6)	
Factor IV (financial health of local government)								
	highest	1,941	(36.9)	254	(13.1)	1,687	(86.9)	0.158
	high	1,110	(21.1)	112	(10.1)	998	(89.9)	
	middle	947	(18.0)	106	(11.2)	841	(88.8)	
	low	633	(12.0)	76	(12.0)	557	(88.0)	
	lowest	628	(11.9)	77	(12.3)	551	(87.7)	

*Results from *χ*^2^ test.

**Except monthly family income (203 missing cases) and tooth-brushing frequency (two missing cases).

DMFT, decayed, missing, and filled teeth index.

Table 2 shows the associations between regional-level characteristics and dental caries experience separately for women and men. Overall, women had a significantly higher (2.25-fold) probability of having dental caries experience than men. The probability of having dental caries experience differed significantly among regional-level characteristics in men, but no such relationship was observed in women. The density of services and medical facilities, dependence on manufacturing industries, and the financial health of local governments were associated with dental caries experience in men. However, no clear linearity was observed in the associations between these factors and dental caries experience.

**Table 2:** Odds ratios of having dental caries experience according to gender

		**Total**		**Women**		**Men**	
		**OR**	**95 % CI**	**OR**	**95 % CI**	**OR**	**95 % CI**
Individual level							
Age (years)		**1.03**	**1.02-1.03**	**1.04**	**1.03-1.05**	**1.02**	**1.01-1.03**
Gender							
	Men	1.00					
	Women	**2.25**	**1.89-2.69**				
Monthly family income (thousand Korean won)							
	≥1500	1.00		1.00		1.00	
	1000–1490	1.07	0.85-1.35	1.21	0.85-1.73	0.97	0.71-1.32
	500–990	1.00	0.80-1.26	1.09	0.77-1.55	0.92	0.68-1.24
	<500	1.13	0.82-1.54	1.06	0.67-1.69	1.13	0.74-1.72
Tooth-brushing frequency (number/day)							
	≥3	1.00		1.00		1.00	
	2	1.00	0.83-1.21	0.88	0.66-1.16	1.09	0.84-1.41
	0–1	1.15	0.80-1.64	0.86	0.45-1.62	1.32	0.85-2.05
Regular dental clinic visits							
	Yes	1.00		1.00		1.00	
	No	**1.40**	**1.07-1.82**	1.34	0.90-1.99	**1.50**	**1.04-2.15**
Regional level							
Factor I (degree of urbanisation)							
	highest	1.00		1.00		1.00	
	high	1.02	0.75-1.39	0.96	0.62-1.48	1.11	0.76-1.60
	middle	1.04	0.75-1.45	0.89	0.56-1.43	1.19	0.79-1.78
	low	1.09	0.76-1.54	1.31	0.75-2.29	0.97	0.64-1.46
	lowest	1.13	0.72-1.77	0.89	0.47-1.71	1.43	0.81-2.54
Factor II (density of services and medical facilities)							
	highest	1.00		1.00		1.00	
	high	0.99	0.70-1.38	0.89	0.55-1.46	1.07	0.71-1.60
	middle	**1.60**	**1.12-2.29**	1.48	0.87-2.51	**1.71**	**1.10-2.66**
	low	1.10	0.77-1.58	0.99	0.59-1.66	1.14	0.73-1.78
	lowest	1.27	0.90-1.80	1.06	0.64-1.74	**1.56**	**1.01-2.39**
Factor III (dependence on manufacturing industry)							
	highest	1.00		1.00		1.00	
	high	0.99	0.69-1.41	1.02	0.60-1.73	0.98	0.63-1.50
	middle	1.14	0.82-1.59	1.15	0.72-1.86	1.11	0.73-1.68
	low	1.38	0.93-2.04	1.32	0.75-2.32	1.41	0.87-2.31
	lowest	**1.51**	**1.09-2.08**	1.51	0.95-2.39	**1.53**	**1.03-2.28**
Factor IV (financial health of local government)							
	highest	1.00		1.00		1.00	
	high	**1.43**	**1.07-1.93**	1.14	0.74-1.74	**1.75**	**1.22-2.52**
	middle	1.22	0.90-1.65	1.07	0.69-1.68	1.29	0.89-1.87
	low	1.19	0.86-1.65	0.88	0.55-1.41	**1.50**	**1.00-2.25**
	lowest	1.11	0.81-1.53	0.92	0.59-1.45	1.32	0.89-1.96

*Statistically significant *p*-values are indicated in bold.

OR, odds ratio; CI, confidence interval.

Some interesting differences were observed in the age-stratified analyses (Table 3). Individual and regional factors showed different patterns and strengths of association across all age groups. Overall, women had a higher odds ratio (OR) of dental caries experience than men in all age groups. People who visited a dental clinic regularly for check-ups had better oral health than those who did not in the 45–55-year age group. In the oldest group (55–64 years), people who brushed their teeth more than three times per day had a lower chance of having dental caries than did those who brushed their teeth twice of less often per day. Regarding regional factors, young adults (19–34 years) living in regions with a mid-level density of services and medical facilities showed a significantly higher OR for dental caries experience than did residents in the highest density region. The effect of factor III (dependence on manufacturing industries) was significant in the young middle-age group (35–44 years) and a significant association between factor IV (financial health of local government) and dental caries experience was observed in the old middle-age group (45–54 years). No significant association was observed in the older age group (55–64 years).

**Table 3:** Odds ratios of having dental caries experience according to age group

		**19–34 years**		**35–44 years**		**45–54 years**		**55–64 years**	
		**OR**	**95 % CI**	**OR**	**95 % CI**	**OR**	**95 % CI**	**OR**	**95 % CI**
Individual level									
Gender									
	Male	1.00		1.00		1.00			
	Women	**1.56**	**1.17-2.07**	**3.00**	**2.16-4.15**	**2.48**	**1.57-3.93**	**2.83**	**1.65-4.84**
Monthly family income (thousand Korean won)									
	≥1500	1.00		1.00		1.00			
	1000–1490	1.00	0.71-1.41	1.23	0.80-1.90	1.04	0.56-1.94	1.51	0.69-3.31
	500–990	0.96	0.67-1.37	0.98	0.63-1.51	0.97	0.53-1.76	1.11	0.57-2.17
	<500	1.03	0.59-1.79	1.09	0.58-2.04	0.70	0.34-1.43	1.66	0.78-3.57
Tooth-brushing frequency (number/day)									
	≥3	1.00		1.00		1.00			
	2	0.84	0.62-1.12	1.03	0.74-1.45	0.85	0.50-1.45	**1.86**	**1.07-3.21**
	0–1	0.64	0.35-1.16	1.19	0.55-2.57	1.01	0.43-2.33	**2.50**	**1.07-5.86**
Regular dental clinic visits									
	Yes	1.00		1.00		1.00			
	No	1.06	0.68-1.64	1.31	0.85-2.03	**1.98**	**1.02-3.83**	2.39	0.90-6.36
Regional level									
Factor I (degree of urbanisation)									
	highest	1.00		1.00		1.00		1.00	
	high	1.11	0.72-1.70	1.21	0.73-2.01	0.92	0.42-2.01	0.65	0.27-1.58
	middle	1.15	0.71-1.84	0.85	0.49-1.46	1.09	0.47-2.54	0.88	0.32-2.43
	low	1.02	0.59-1.76	0.91	0.50-1.68	1.34	0.56-3.21	1.02	0.41-2.50
	lowest	0.97	0.42-2.22	1.12	0.49-2.53	0.92	0.34-2.54	0.98	0.34-2.82
Factor II (density of services and medical facilities)									
	highest	1.00		1.00		1.00		1.00	
	high	1.06	0.65-1.72	1.17	0.64-2.16	0.91	0.40-2.06	0.51	0.21-1.27
	middle	**1.93**	**1.14-3.30**	1.26	0.68-2.35	1.93	0.76-4.94	1.13	0.41-3.10
	low	1.07	0.63-1.81	1.29	0.70-2.39	1.08	0.42-2.82	0.85	0.30-2.38
	lowest	1.42	0.87-2.32	1.42	0.79-2.55	1.19	0.47-3.04	0.63	0.23-1.75
Factor III (dependence on manufacturing industry)									
	highest	1.00		1.00		1.00		1.00	
	high	0.80	0.47-1.38	1.42	0.78-2.60	1.02	0.41-2.57	0.79	0.30-2.10
	middle	1.37	0.82-2.29	0.85	0.50-1.44	1.19	0.50-2.82	1.28	0.50-3.26
	low	1.18	0.66-2.10	1.62	0.82-3.21	1.46	0.54-3.97	1.22	0.39-3.76
	lowest	1.32	0.84-2.05	**2.01**	**1.14-3.57**	1.56	0.64-3.81	1.03	0.40-2.68
Factor IV (financial health of local government)									
	highest	1.00		1.00		1.00		1.00	
	high	1.56	1.00-2.43	1.47	0.88-2.46	1.57	0.78-3.17	1.13	0.51-2.48
	middle	0.99	0.65-1.52	1.28	0.75-2.17	**2.47**	**1.02-5.97**	1.23	0.54-2.79
	low	1.08	0.67-1.76	0.99	0.58-1.71	**2.63**	**1.05-6.56**	0.88	0.38-2.04
	lowest	1.11	0.71-1.73	1.21	0.67-2.19	1.00	0.46-2.18	1.25	0.50-3.12

*Statistically significant *p*-values are indicated in bold.

OR, odds ratio; CI, confidence interval.

## Discussion

In this study, we examined the associations between different aspects of the neighbourhood environment and dental caries experience according to gender and age. The results showed that the regional socio-economic context was linked with caries in men and that the associations between regional contextual variables and dental caries experience differed by age group, consistent with the findings of several previous studies [14,15]. Although many studies have reported significant effects of the socio-economic context on dental caries experience, most studies have used global proxies, such as rurality or deprivation, to describe the regional socio-economic environment [2,5-9]. However, recent thinking on the role of social and material environments in generating health inequalities has led to the collection of true ‘area’ data that accurately reflect the character, amenableness, and opportunity afforded by the area or neighbourhood in which people live [16,17]. In this study, we constructed new regional contextual variables from various socio-economic indices that reflected different aspects of the regions using factor analysis.

Traditionally, the social context has been shown to have a greater influence on women than on men because they spend more time at home looking after children, doing domestic work, or being primary caregivers [18-22]. In contrast, the results of our study indicated that the regional context was relatively beneficial in reducing the prevalence of caries experience in men; however, this effect was weaker than that observed for women. The association between neighbourhood disadvantage and poor health is not always straightforward [23]. Some evidence has suggested that the place of residence may not affect all people in the same way [23-27]. Time spent at home seems an unlikely explanation, given the lack of a substantial gender difference in exposure to the residential environment [22]. Studies that have specifically explored men and women with similar working, social, and material circumstances have shown a reduction in, or the absence of, gender-based morbidity [14,28]. Indeed, the inconsistency may be due to differences in the conceptualisation and measurement of the social environment. Because we focused primarily on regional factors, such as material, physical, and economic capital, men’s health status could have been more affected by regional context. However, some studies that measured perceived social context, such as social support networks, social capital, social participation, or trust, found that it had a greater influence on women than on men [29-32].

The conceptual distinction between biological sex and social gender is also important in efforts to better understand the oral health difference between men and women. However, the effects of these characteristics may be difficult to disentangle in specific contexts [13]. Gender is a dynamic set of socially constructed relationships embedded in everyday interactions, rather than a simple individual attribute [33]. Many studies have shown that caries experience rates are higher in women than in men [12,13,15]. These variations have traditionally been assumed to be biological in origin, but recent work has demonstrated that these effects may be due to multi-factorial behavioural issues involving both sex and gender [13]. Social aspects that require further investigation include gender differences in diet and eating patterns, as well as wider dimensions of inequality between women and men [12,13,34].

In this study, we showed that residents of regions with mid-level services and medical facilities had a higher risk of having dental caries experience than did those of regions with the highest level of facilities, after controlling for individual-level variables; in age-stratified analyses, this association was observed only in young adults (19–34 years). Public and private services provided to support people in their daily lives, including medical services, are one of the most commonly proposed regional socio-economic factors that affect the health of residents. However, little evidence is available to support the hypothesis that an increase in a region’s health resources directly affects residents’ health status. McIntyre and Ellaway [35] conceptualised regional features, including features aggregated in factor II in this study, as ‘opportunity structures’; that is, socially constructed and socially patterned features of the physical and social environments that may promote or damage health, directly or indirectly, through the possibilities they provide for people to live healthy lives. The Korean government is currently collecting small-area dental service statistics, such as dentist- to-population ratios and numbers of clinics, and we expect that studies conducted in the near future will benefit from the use of such statistics.

Residents of regions that were least dependent on manufacturing industries exhibited a significantly higher prevalence of dental caries than those of the most dependent regions. Age-stratified analyses showed a significant association only in the young middle-age group (35–44 years). Socio-economic factors affecting health status could operate positively or negatively, and these factors did not show the same effects in all areas [36]. The positive and negative aspects of the manufacturing industry may directly or indirectly influence individual health status through different mechanisms and magnitudes, according to the degree of dependence. Regarding factor IV, which reflects the affluence of local government, people aged 45–54 years living in regions with mid–low levels of affluence showed a significantly higher risk of dental caries experience, but those in the poorest regions did not. This nonlinear relationship also indicates that the socio-economic factors affecting the health status of residents operate through different mechanisms or degrees, according to geographic location.

The last decade has seen a growing interest in the links among sex, gender, and health, and the importance of these issues has been recognised not only from the perspective of equity, but also as a prerequisite for more effective care [13]. As a result, a large body of evidence based on differences in patterns of morbidity and mortality between women and men has accumulated. However, these issues have received little attention to date in the context of oral health research and practice. The fact that men and women interact with their environment in a different way, and thus are likely to benefit from different types of intervention, may also have important health policy implications. However, further research is needed to gain a more detailed understanding of the social processes underlying these gender health effects [19].

In this study, age seemed to act as a possible effect modifier of environmental impact on dental caries experience, although this was not obvious from our data. Based on the results of the present study, we suggest that young adults (19–34 years) living in areas with good services and medical facilities were less susceptible to having dental caries experience. Likewise, old middle-aged (45–54 years) people had a higher risk of having dental caries experience if they lived in regions with a financially weak local government. This finding may be because the comprehensiveness of dental health services provided by community health care centres is highly dependent on the financial status of the local government in Korea, and these services are usually used by older people rather than younger individuals, who tend to have less interest in community affairs. A full assessment of this issue requires more clear evidence and the establishment of a causal background. However, these various age-related patterns can provide meaningful information about complex environmental factors that could affect individual dental caries experience by age group in different ways.

This study has some limitations. First, the cross-sectional nature of the data used in this analysis did not allow us to investigate the directionality of the associations or to clarify the time frame of the exposures. Second, we did not measure the length of time that participants had spent in their neighbourhoods or the extent of their exposure to the neighbourhood environment. Third, our use of the combined DMFT index as an outcome in this study did not allow us to separate the proportions of caries, missing, and filled teeth present, which could be affected in different ways by the neighbourhood environment during the study period from that during the subjects’ lives as a whole. Indeed, filled and missing teeth are indicators of having once experienced caries. However, with the ageing process, tooth loss could also be associated generally with periodontal disease. Nonetheless, the DMFT index remains the basis for caries measurement and has important epidemiological significance [37-40]. Fourth, we investigated the association between regional context and dental caries experience according to gender and age groups. Because we performed multiple subgroup analyses, the probability of a false positive finding should be considered [41]. Finally, our results may have been affected be residual confounding by other behavioural and regional characteristics related to caries experience, such as sugar-related dietary habits and oral hygienic status, including the use of fluoride toothpastes and mouth rinses. Water fluoridation is an important issue affecting caries-related community factors, but Korean National Oral Health Survey (KNOHS) data do not include such information; however, the coverage rate is very low. Additionally, we did not obtain health insurance information because the Korean government’s national health insurance system covers almost all Koreans. Although some preventative and aesthetic dental treatments are not covered, basic treatment for dental caries, such as dental filling, is covered by national health insurance. Overall, further studies are needed to address the limitations of the present study.

## Conclusions

The findings of this study indicate that socio-economic factors affecting the dental health status of residents may operate through different mechanisms or degrees according to geographic location, suggesting that some age and gender subgroups are likely to benefit from different types of intervention, including the development of specific health policies.

## Methods

### Data source and study population

Data on dental caries and individual characteristics for this study were obtained from the KNOHS of 2000 [42]. This survey included 21,829 people who were selected as a representative sample of the Korean household population aged ≥ 2 years. Of these, 5,259 participants >19 years and <65 years of age completed an oral health examination and an interview-based questionnaire.

### Dependent variables

To measure the dental caries experience, the DMFT index was recorded by trained dentists according to the World Health Organization epidemiologic survey guidelines for oral health [43]. Individuals with DMFT index scores ≥ 1 were classified as having dental caries experience.

### Individual-level variables

Information on individual characteristics was collected by trained interviewers using standardised questionnaires, including demographic factors (age, gender), health behaviour (tooth-brushing frequency per day, frequency of regular dental check-ups), and socio-economic factors (average monthly household income).

### Regional-level variables

Regional characteristics were retrieved from the “Major statistical indices of Si-Gun-Gu” (city-county-ward) published by the Korean Statistical Office [44], which contained 81 indices reflecting regional demographic structure, services, and social and material infrastructure in 10 categories for all 232 basic administrative Si-Gun-Gu districts in Korea. In this study, 31 indices from 1998 in eight categories were selected as our basic regional-level data. As anticipated, many of these variables were interrelated; thus, exploratory factor analysis was conducted using the SAS software (ver. 9.1), adopting the principal component method and orthogonal varimax rotation. Five factors with eigenvalues > 1.0 were identified and, of these, the first four with higher eigenvalues and theoretical coherence were selected. These four factors accounted for 76.9 % of the variability in our original regional indices (Table 4).

**Table 4:** Regional socio-economic indices and rotated factor patterns

**Index**		**Factor I**	**Factor II**	**Factor III**	**Factor IV**
Population					
	Registered population	0.70630	−0.04509	−0.03846	0.59089
	Persons/square kilometre	0.62011	0.21722	−0.08753	−0.01431
	No. of births per 1,000 persons	0.67764	−0.20903	0.37741	0.17307
	No. of deaths per 1,000 persons	−0.95147	−0.09328	−0.10155	−0.16511
	No. of marriages per 1,000 persons	0.71852	0.03687	0.33266	0.11668
	No. of divorces per 1,000 persons	0.76403	0.20042	0.18045	−0.08721
	No. moving in per 1,000 persons	0.81729	0.07612	−0.01002	0.20400
	No. moving out per 1,000 persons	0.83756	0.24285	−0.06981	0.09914
	Percent of elderly people (≥65 years)	−0.94515	−0.06809	−0.13560	−0.11916
Agriculture					
	No. of farming households /all households	−0.76057	−0.20671	0.07179	0.38994
	No. of farmers /registered population	−0.70063	−0.20574	0.11421	0.42263
	Per capita area under cultivation	−0.89877	−0.18861	−0.06235	−0.04281
Manufacturing Industry					
	No. of manufacturing companies per 1,000 persons	0.04816	0.29067	0.81271	−0.04365
	No. of manufacturing workers/registered population	0.09585	−0.02411	0.95265	0.01426
	Per capita production from manufacturing industries	0.08634	−0.10685	0.78910	0.08927
Housing/Construction					
	No. of townhouses/all homes	−0.91915	−0.00569	−0.05562	−0.21297
	No. of apartments/all homes	0.84051	−0.04889	−0.01530	0.26510
	No. of tenement houses/all homes	0.65028	−0.0021	0.18450	0.03346
	No. of owner-occupied homes/all homes	−0.91408	−0.25359	−0.05341	−0.03496
	No. of deposit-basis leased homes/all homes	0.88661	0.19355	0.04016	0.09788
	No. of monthly rent homes/all homes	0.72684	0.28249	0.02696	0.01139
	Per capita length of roads	−0.74692	−0.12811	−0.12375	−0.25597
Transportation					
	No. of cars per 100 persons	0.30838	0.31150	0.40635	0.18867
	No. of privately owned cars per 100 persons	0.70451	0.24995	0.26636	0.28362
Service Industry					
	No. of banks per 1,000 persons	0.18572	0.89966	0.13660	0.06476
	No. of restaurants per 1,000 persons	−0.09718	0.80765	0.15243	−0.16288
Health					
	Number of hospitals and clinics per 1,000 persons	0.33628	0.83723	−0.07375	0.06638
	Number of physicians per 1,000 persons	0.18359	0.86048	−0.05135	0.02972
Public Finance					
	Amount of local taxes collected	0.54397	0.36858	0.09104	0.62067
	Expenditure from general account	0.12103	−0.04679	0.01980	0.88998
	Financial independency rate	0.72692	0.24522	0.23032	0.45656
	Eigenvalue*	15.30554	3.37322	3.20474	1.962914
	Proportion*	0.49370	0.10880	0.10340	0.06330
	Cumulative*	0.49370	0.60250	0.70590	0.76920

*Results from factor analysis.

These factors were conceptualised from the factor loadings of each index as ‘degree of urbanisation (factor I)’, ‘density of service and medical facilities (factor II)’, ‘dependence on the manufacturing industry (factor III)’, and ‘financial health of local government (factor IV)’. Scores for each factor were calculated for all districts, and the districts were then ranked in descending order using these scores. All districts were categorised into quintile groups by rank: 1) highest, 2) high, 3) middle, 4) low, and 5) lowest. These quintile groups were used as regional-level variables in factor analyses, and were attached to each of the 5,259 individuals according to residential area (118 Si-Gun-Gu districts).

### Data analysis

A chi-squared test was conducted to evaluate the distributions of individual characteristics and dental caries experience according to regional factor groups using the SAS software (ver. 9.1). To allow for the non-independence of study subjects clustered within districts, a two-level random intercept logistic regression model was fitted to the dental caries experience data using MLwiN (ver. 2.02), adjusting for individual- and regional-level variables. Second-order PQL estimates were obtained from the models. To examine differences in the effects of regional variables according to gender and age groups, we stratified the sample into women and men, and into four 10-year-interval age groups (19–34, 35–44, 45–54, and 55–64 years), and the same model was used for each subgroup.

## Competing interests

The authors declare that they have no competing interest.

## Author contributions

HYL wrote the draft. YHC and HWP collected and analysed the data. SGL contributed to the conception of the study and interpreted the results with YHC. All authors read and approved the final manuscript.

## References

[B1] Bagramian RA Garcia Godoy F Volpe AR The global increase in dental caries. A pending public health crisis Am J Dent 2009 22 1 3 8 19281105

[B2] Tellez M Sohn W Burt BA Ismail AI Assessment of the relationship between neighborhood characteristics and dental caries severity among low-income African-Americans: a multilevel approach J Public Health Dent 2006 66 1 30 36 16570748 10.1111/j.1752-7325.2006.tb02548.x PMC1817893

[B3] Choi Y Lee SG Does regional socioeconomic context affect the dental caries experience? A multilevel study of Korean adults Eur J Oral Sci 2011 119 4 294 300 21726290 10.1111/j.1600-0722.2011.00831.x

[B4] Holst D Schuller AA Aleksejunien J Eriksen HM Caries in populations–a theoretical, causal approach Eur J Oral Sci 2001 109 3 143 148 11456342 10.1034/j.1600-0722.2001.00022.x

[B5] Levin KA Davies CA Douglas GV Pitts NB Urban–rural differences in dental caries of 5-year old children in Scotland Soc Sci Med 2010 71 11 2020 2027 20965633 10.1016/j.socscimed.2010.09.006

[B6] Levin KA Davies CA Topping GV Assaf AV Pitts NB Inequalities in dental caries of 5-year-old children in Scotland, 1993–2003 Eur J Public Health 2009 19 3 337 342 19307245 10.1093/eurpub/ckp035

[B7] Aida J Ando Y Oosaka M Niimi K Morita M Contributions of social context to inequality in dental caries: a multilevel analysis of Japanese 3-year-old children Community Dent Oral Epidemiol 2008 36 2 149 156 18333879 10.1111/j.1600-0528.2007.00380.x

[B8] Antunes JLF Peres MA Mello TRC Waldman EA Multilevel assessment of determinants of dental caries experience in Brazil Community Dent Oral Epidemiol 2006 34 2 146 152 16515679 10.1111/j.1600-0528.2006.00274.x

[B9] Pattussi MP Hardy R Sheiham A The potential impact of neighborhood empowerment on dental caries among adolescents Community Dent Oral Epidemiol 2006 34 5 344 350 16948673 10.1111/j.1600-0528.2006.00283.x

[B10] Michael Marmot RGW Social determinants of health 2000 Oxford University, New York

[B11] Madlena M Hermann P Jahn M Fejerdy P Caries prevalence and tooth loss in Hungarian adult population: results of a national survey BMC Publ Health 2008 8 1 364 10.1186/1471-2458-8-364 PMC2596128 18939981

[B12] Lukacs J Sex differences in dental caries experience: clinical evidence, complex etiology Clin Oral Investig 2011 15 5 649 656 10.1007/s00784-010-0445-3 20652339

[B13] Doyal L Naidoo S Why dentists should take a greater interest in sex and gender Br Dent J 2010 209 7 335 337 20930773 10.1038/sj.bdj.2010.883

[B14] Lundegren N Axtelius B Åkerman S Oral health in the adult population of Skåne, Sweden: a clinical study Acta Odontol Scand 2012 1 9 Epub ahead of print 22181829 10.3109/00016357.2011.640279

[B15] Maria F Alexandre RV Explaining gender differences in caries: a multifactorial approach to a multifactorial disease Int J Dent 2010 649643 1 5 10.1155/2010/649643 PMC2840374 20339488

[B16] Macintyre S Ellaway A Cummins S Place effects on health: how can we conceptualise, operationalise and measure them? Soc Sci Med 2002 55 1 125 139 12137182 10.1016/s0277-9536(01)00214-3

[B17] Cummins S Macintyre S Davidson S Ellaway A Measuring neighbourhood social and material context: generation and interpretation of ecological data from routine and non-routine sources Health Place 2005 11 3 249 260 15774331 10.1016/j.healthplace.2004.05.003

[B18] Chuang Y Chuang K Gender differences in relationships between social capital and individual smoking and drinking behavior in Taiwan Soc Sci Med 2008 67 8 1321 1330 18667260 10.1016/j.socscimed.2008.06.033

[B19] Poortinga W Dunstan FD Fone DL Perceptions of the neighbourhood environment and self rated health: a multilevel analysis of the Caerphilly Health and Social Needs Study BMC Publ Health 2007 7 285 10.1186/1471-2458-7-285 PMC2100049 17925028

[B20] Skrabski Á Kopp M Kawachi I Social capital in a changing society: cross sectional associations with middle aged female and male mortality rates J Epidemiol Community Health 2003 57 2 114 119 12540686 10.1136/jech.57.2.114 PMC1732386

[B21] Kavanagh AM Bentley R Turrell G Broom DH Subramanian SV Does gender modify associations between self rated health and the social and economic characteristics of local environments? J Epidemiol Community Health 2006 60 6 490 495 16698978 10.1136/jech.2005.043562 PMC2563944

[B22] Stafford M Cummins S Macintyre S Ellaway A Marmot M Gender differences in the associations between health and neighbourhood environment Soc Sci Med 2005 60 8 1681 1692 15686801 10.1016/j.socscimed.2004.08.028

[B23] Yen IH Michael YL Perdue L Neighborhood environment in studies of health of older adults: a systematic review Am J Prev Med 2009 37 5 455 463 19840702 10.1016/j.amepre.2009.06.022 PMC2785463

[B24] Stafford M Marmot M Neighbourhood deprivation and health: does it affect us all equally? Int J Epidemiol 2003 32 3 357 366 12777420 10.1093/ije/dyg084

[B25] Fone DL Dunstan F Mental health, places and people: A multilevel analysis of economic inactivity and social deprivation Health Place 2006 12 3 332 344 16546698 10.1016/j.healthplace.2005.02.002

[B26] Fone D Lloyd K Dunstan F Measuring the neighbourhood using UK benefits data: a multilevel analysis of mental health status BMC Publ Health 2007 7 1 69 10.1186/1471-2458-7-69 PMC1878475 17477868

[B27] Fone D Dunstan F Williams G Lloyd K Palmer S Places, people and mental health: A multilevel analysis of economic inactivity Soc Sci Med 2007 64 3 633 645 17070974 10.1016/j.socscimed.2006.09.020

[B28] Lohan M How might we understand men's health better? Integrating explanations from critical studies on men and inequalities in health Soc Sci Med 2007 65 3 493 504 17559996 10.1016/j.socscimed.2007.04.020

[B29] Chuang Y-C Li Y-S Wu Y-H Chao H A multilevel analysis of neighborhood and individual effects on individual smoking and drinking in Taiwan BMC Publ Health 2007 7 1 151 10.1186/1471-2458-7-151 PMC1955439 17623053

[B30] Bower E Gulliford M Steele J Newton T Area deprivation and oral health in Scottish adults: a multilevel study Community Dent Oral Epidemiol 2007 35 2 118 129 17331153 10.1111/j.1600-0528.2007.00308.x

[B31] Newton JT Bower EJ The social determinants of oral health: new approaches to conceptualizing and researching complex causal networks Community Dent Oral Epidemiol 2005 33 1 25 34 15642044 10.1111/j.1600-0528.2004.00190.x

[B32] Molinari C Ahern M Hendryx M The relationship of community quality to the health of women and men Soc Sci Med 1998 47 8 1113 1120 9723856 10.1016/s0277-9536(98)00114-2

[B33] Emslie C Hunt K The weaker sex? Exploring lay understandings of gender differences in life expectancy: a qualitative study Soc Sci Med 2008 67 5 808 816 18558455 10.1016/j.socscimed.2008.05.009 PMC2570197

[B34] Lukacs JR Largaespada LL Explaining sex differences in dental caries prevalence: Saliva, hormones, and “life-history” etiologies Am J Hum Biol 2006 18 4 540 555 16788889 10.1002/ajhb.20530

[B35] MCINTYRE SEA BERKMAN LF KI Ecological approaches: rediscovering the role of the physical and social environment Social epidemiology 2000 Oxford University, New York 332 348

[B36] Curtis S Rees Jones I Is There a Place for Geography in the Analysis of Health Inequality? Sociol Health Illn 1998 20 5 645 672

[B37] Schuller AA Holst D Oral status indicators DMFT and FS-T: reflections on index selection Eur J Oral Sci 2001 109 3 155 159 11456344 10.1034/j.1600-0722.2001.00016.x

[B38] Benigeri M Payette M Brodeur J-M Comparison between the DMF indices and two alternative composite indicators of dental health Community Dent Oral Epidemiol 1998 26 5 303 309 9792121 10.1111/j.1600-0528.1998.tb01965.x

[B39] Campus G Sacco G Cagetti M Abati S Changing trend of caries from 1989 to 2004 among 12-year old Sardinian children BMC Publ Health 2007 7 1 28 10.1186/1471-2458-7-28 PMC1832181 17331258

[B40] Bernabé E Suominen-Taipale AL Vehkalahti MM Nordblad A Sheiham A The T-Health index: a composite indicator of dental health Eur J Oral Sci 2009 117 4 385 389 19627349 10.1111/j.1600-0722.2009.00649.x

[B41] Wang R Lagakos SW Ware JH Hunter DJ Drazen JM Statistics in Medicine — Reporting of Subgroup Analyses in Clinical Trials N Engl J Med 2007 357 21 2189 2194 18032770 10.1056/NEJMsr077003

[B42] Ministry of Health and Welfare Korean National Oral Health Survey 2000 1 Ministry of Health and Welfare, Seoul, Korea

[B43] World Health Organization Oral Health Surveys Basic Methods 1997 4 World Health Organiztion, Geneva

[B44] Korean Statistical Office Major statistical indices of Si-Gun-Gu 2000 Korean Statistical Office, Seoul, Korea

